# A new genus and species of pennatulacean octocoral from equatorial West Africa (Cnidaria, Anthozoa, Virgulariidae)

**DOI:** 10.3897/zookeys.546.6344

**Published:** 2015-12-16

**Authors:** Gary C. Williams

**Affiliations:** 1 Department of Invertebrate Zoology and Geology, California Academy of Sciences, 55 Music Concourse Drive, San Francisco, California 94118, USA

**Keywords:** Pennatulacea, Virgulariidae, sea pens, new genus and species, Gulf of Guinea, West Africa, Nigeria, key to the virgulariid genera

## Abstract

A new genus and species of sea pen or virgulariid pennatulacean from the Gulf of Guinea in the tropical eastern Atlantic is described, and a key to the genera of the Virgulariidae is included. The new genus and species described here adds to the previously described five other genera of the family. It is distinguished by unique sclerite and polyp leaf characters from the superficially-similar genus *Virgularia*, which lacks conspicuous sclerites in the polyp leaves and coenenchyme (other than minute oval bodies that are generally <0.01 mm in length).

## Introduction

The biogeographic region of the Mediterranean Sea and Atlantic coast of Africa is home to several apparently endemic octocoral genera, including the pennatulaceans *Amphibelemnon* López-González, Gili & Williams, 2000 and *Crassophyllum* Tixier-Durivault, 1961 (Williams 2011: 4, fig. 2), as well as the alcyonacean *Nidaliopsis* Kükenthal, 1906 (Verseveldt and Bayer 1988: 63–66). Four colonies of a third such pennatulacean has recently been revealed after over thirty years of storage in a museum marine invertebrate collection. These colonies from the Niger River Delta of Nigeria represent the only known specimens from the Niger River delta of a previously undescribed genus and species of sea pen.

Five genera were previously included in the family Virgulariidae (Williams, 1995: 122–125) – *Acanthoptilum* Kölliker, 1870; *Scytaliopsis* Gravier, 1906; *Scytalium* Herklots, 1858; *Stylatula* Verrill, 1864; and *Virgularia* Lamarck, 1816. The new genus described here adds an additional genus. Consequently, a total of six genera are here recognized as comprising the family Virgulariidae.

Virgulariid sea pens are known to range from intertidal habitats to approximately 1200 m in depth (Williams 2011: 6, Fig. 4). The ecological importance of sea pens has recently been increasingly recognized. Baillon et al. (2012) have shown that at least several species of deep-water pennatulaceans in the northwest Atlantic – including *Anthoptilum grandiflorum* (Verrill, 1879) and *Pennatula aculeata* Danielssen, 1860 – can act as nurseries for larval fish such as *Sebastes* spp., or can provide habitat for other fish species. In regards to virgulariid sea pens, often abundant constituents of endangered, impacted or protected soft bottom habitats such as *Stylatula elongata* (Gabb, 1863) in San Francisco Bay and the estuary regions of central California (Mooi et al. 2007: 27). Behavioral observations of species in the genus *Virgularia* regarding their unique ability of rapidly withdrawing the entire colony into the soft substratum, have been recorded for over 400 years. Darwin (1845: 98–100) describes his observation of the withdrawal and reappearance of a Patagonian species, and refers to a similar observation by Captain James Lancaster in Indonesia in 1601. Likewise, Ambroso, et al. (2013) describe their in situ observations of withdrawal behavior in *Virgularia mirabilis*.

It is the aim of this paper to describe a new genus and species of pennatulacean octocoral previously unknown to science, to name the new genus in recognition of the significant career contributions of a prominent octocoral systematist, and to differentiate the new genus from all other genera in the family based on morphological comparisons.

## Materials and methods

Material for this study was revealed during a survey of the pennatulacean octocoral collection at the Museum Support Center (MSC), Smithsonian Institution, Washington DC (National Museum of Natural History, Department of Invertebrate Zoology) in April of 2013. An examination of the material showed that it represented a previously-undescribed genus and species of sea pen. The material was processed as a loan, and laboratory work was conducted at the California Academy of Sciences, San Francisco.

Scanning electron micrographs were made in the Academy’s SEM laboratory with a LEO 1450 VP scanning electron microscope after coating the sclerites on a standard SEM pin stub mount (12.7 mm in diameter and 8 mm pin height) with gold/palladium.

### Abbreviations used in text

USNM United States National Museum

NMNH National Museum of Natural History, Smithsonian Institution, Washington, D.C.

CASIZ California Academy of Sciences Invertebrate Zoology, San Francisco, California

## Systematic account

### Family Virgulariidae Verrill, 1868

#### Genus Grasshoffia gen. n.

Taxon classificationAnimaliaPennatulaceaVirgulariidae

http://zoobank.org/913459D9-D166-4A6E-B998-F8991C19BED4

**Figure 1.**
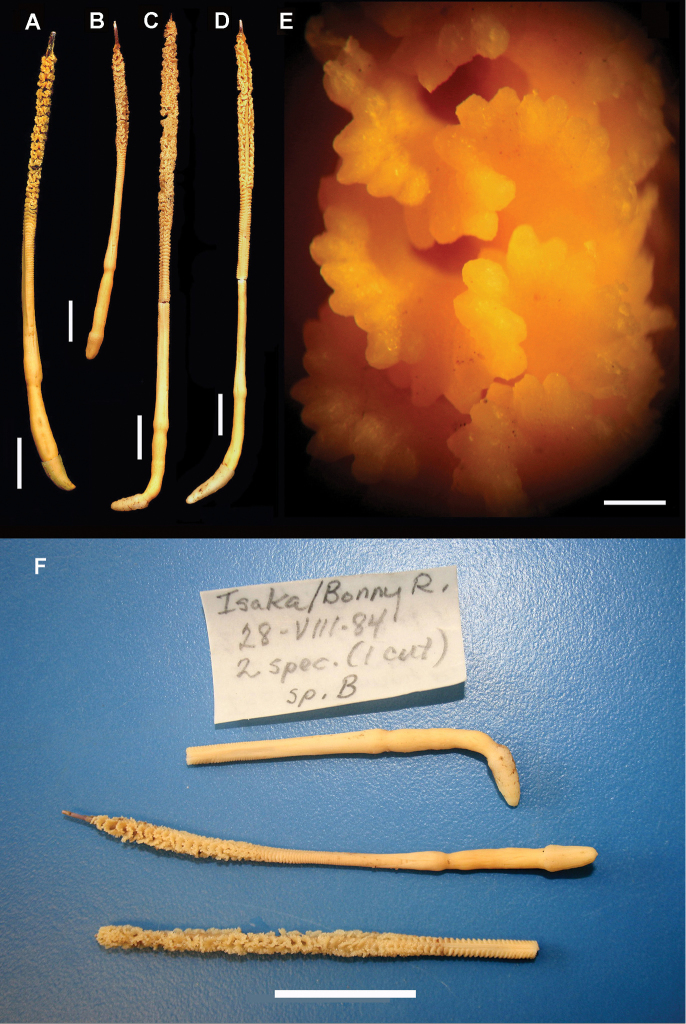
*Grasshoffia virgularioides* gen. & sp. n. External morphology. **A** Holotype (USNM 1205583) **B** Paratype 2 (USNM 1231550) **C** Paratype 1 (USNM 1231549) **D** Paratype 3 (USNM 1205580) **E** Detail of rachis of holotype showing polyps on convoluted polyp leaves **F** Material from the Smithsonian’s Museum Support Center invertebrate zoology collections. Top, USNM 1231549, Paratype 1 (peduncle); Middle, USNM 1231550, Paratype 2 (entire specimen); Bottom: USNM 1231549, Paratype 1 (rachis). Scale bar: 20 mm (**F**); 0.5 mm (**E**); 20 mm (**F**); 10 mm (**A–D**).

**Figure 2.**
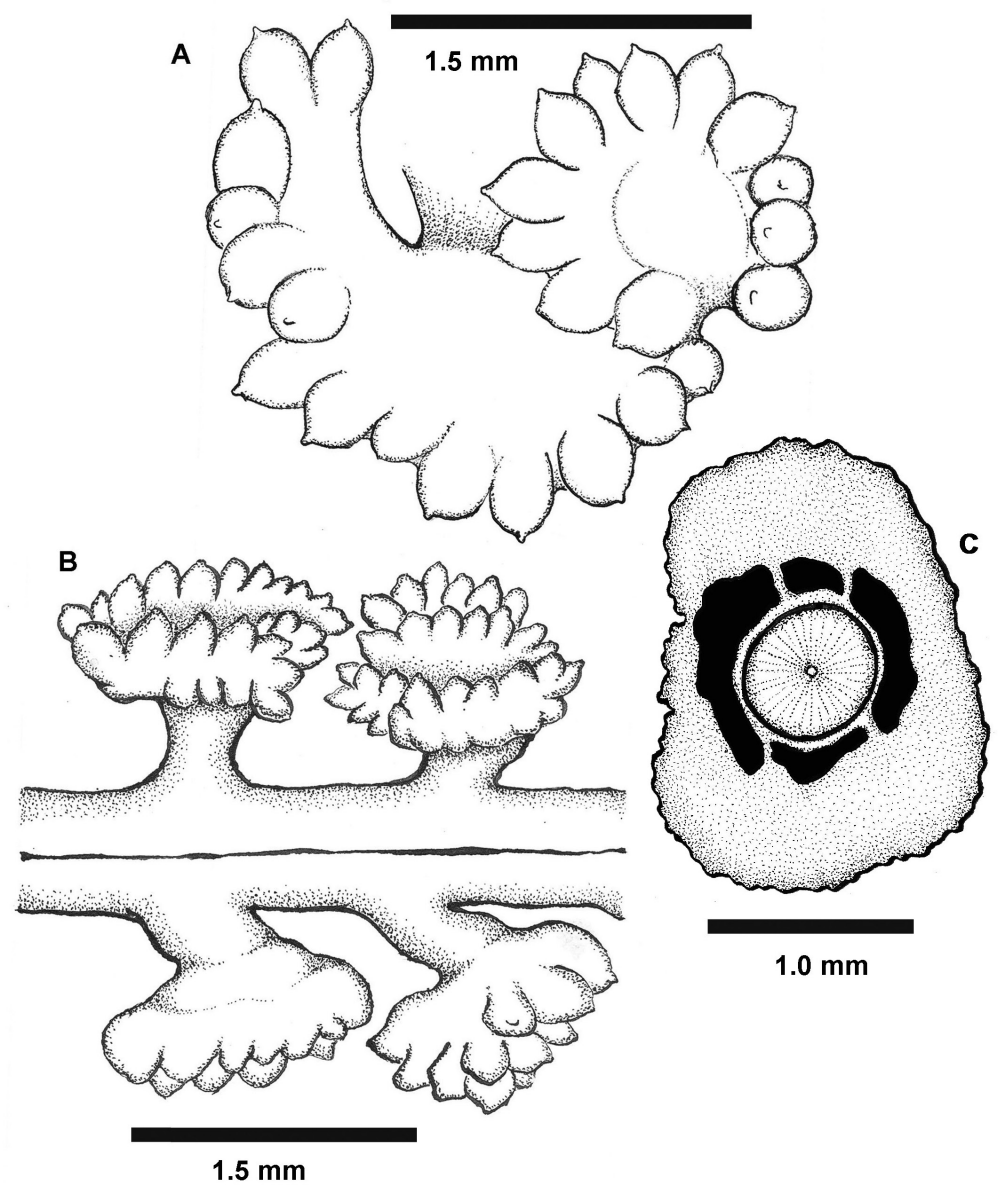
*Grasshoffia virgularioides* gen. & sp. n. Diagrams of polyp leaves from the holotype. **A** A single polyp leaf showing convoluted overall shape and numerous, bulbous, retracted polyps with acute apical tips **B** Dorsal side of the rachis showing congested placement of polyps on sub-circular polyp leaves attached to the rachis by narrow, neck-like stalks **C** Transverse section of paratype 1 (USNM 1231549) at the proximal-most level of the rachis, showing the circular axis and four surrounding longitudinal canals. Scale bar: 1.0 mm (**C**); 1.5 mm (**B**).

**Figure 3.**
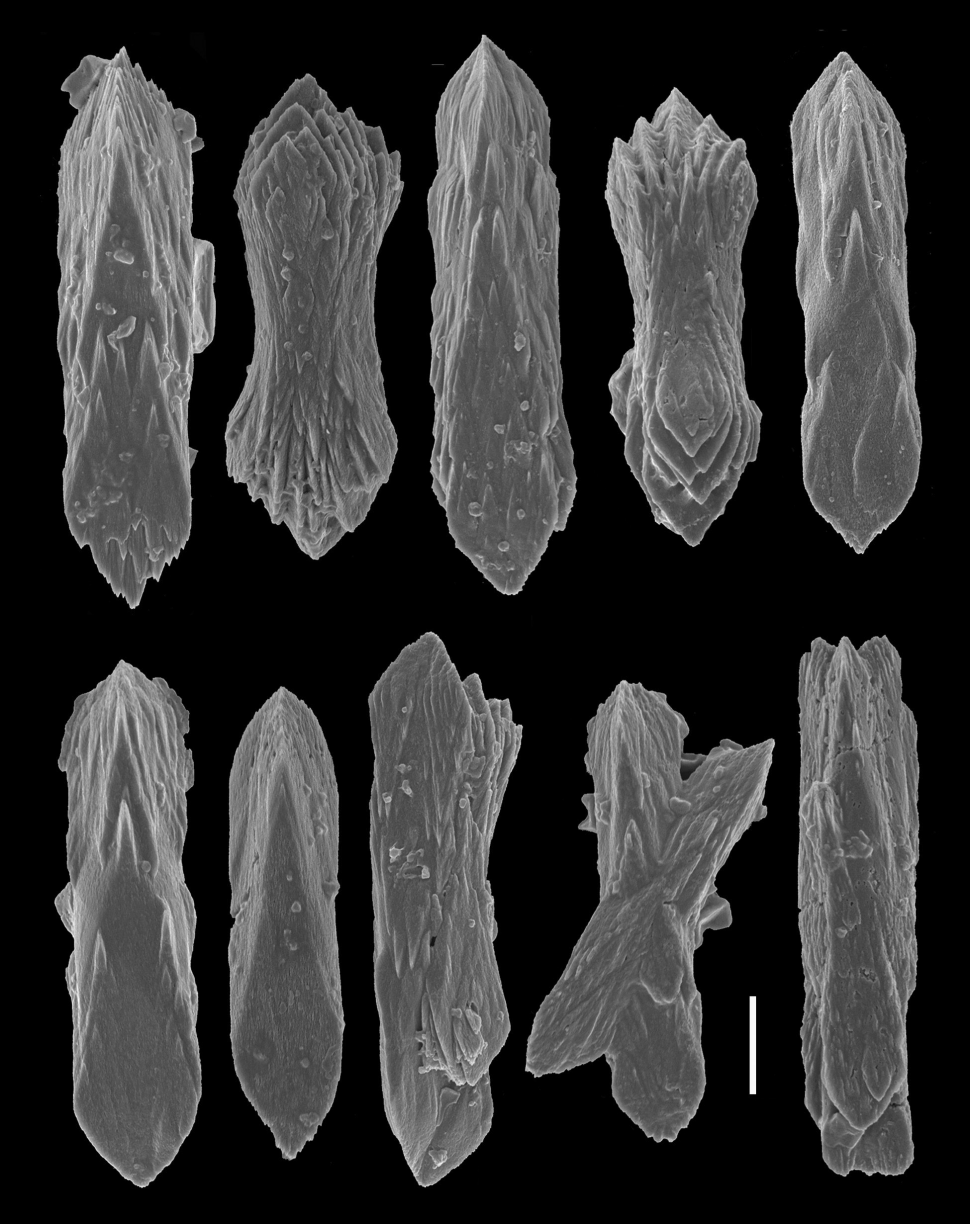
*Grasshoffia virgularioides* gen. & sp. n. Scanning electron micrographs of sclerites from a polyp leaf and polyp walls of the holotype. Scale bar: 0.01 mm.

**Figure 4.**
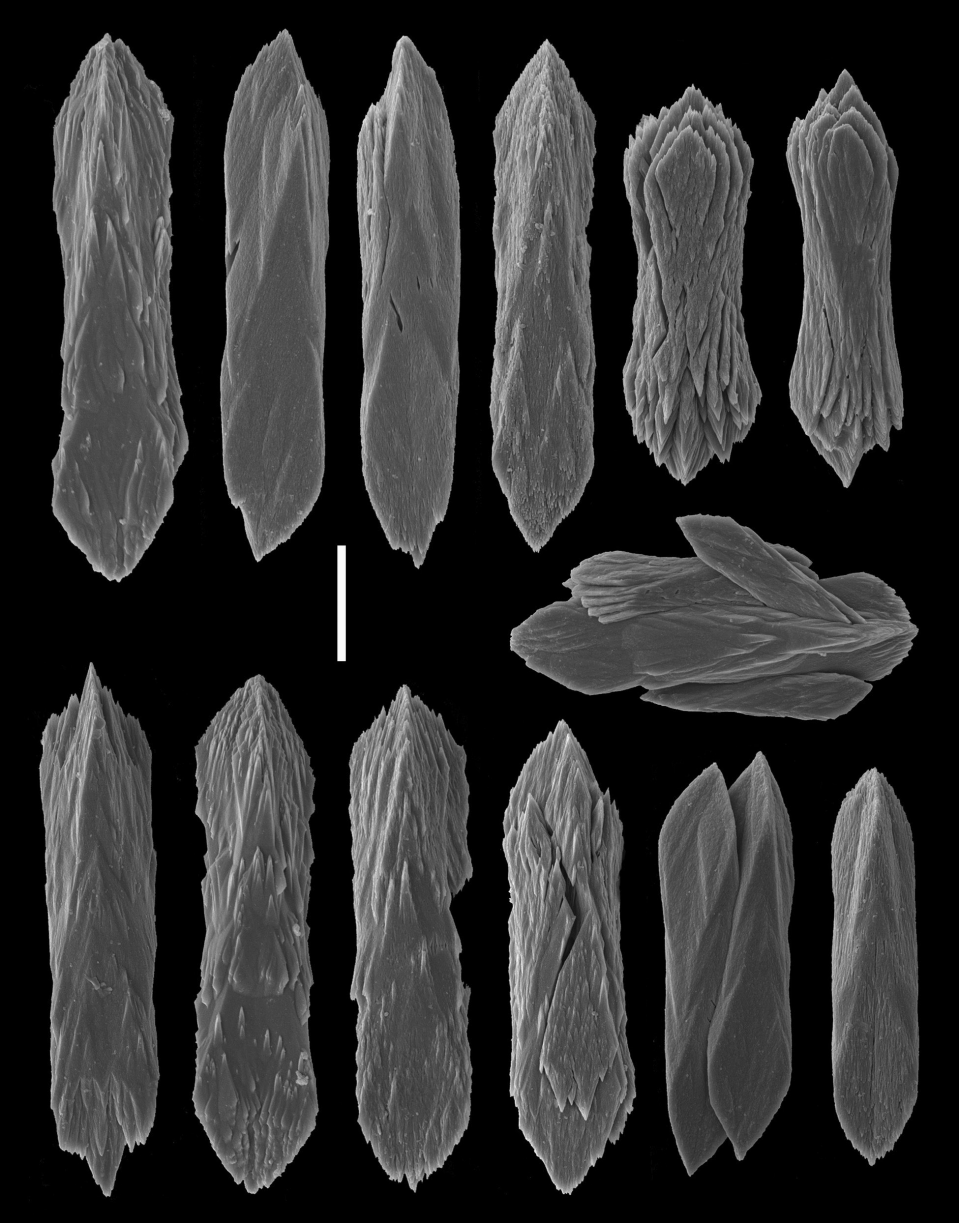
*Grasshoffia virgularioides* gen. & sp. n. Scanning electron micrographs of peduncle sclerites from the holotype. Scale bar: 0.01 mm.

**Figure 5.**
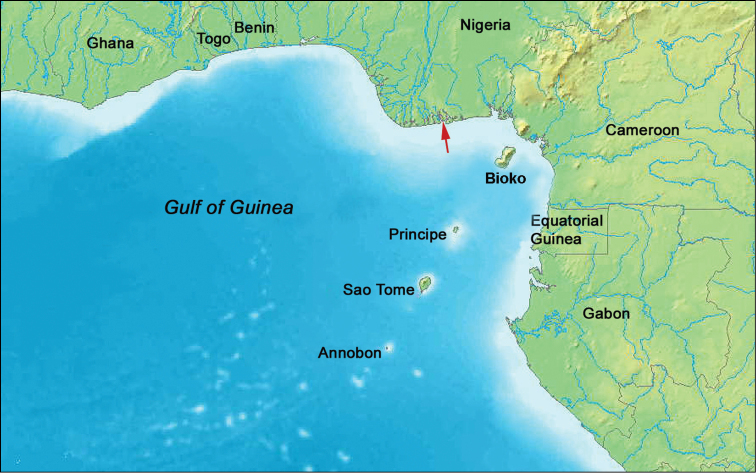
Map of the Gulf of Guinea, West Africa, showing type locality of *Grasshoffia virgularioides* gen. and sp. n. (arrow).

Figures 1 , 2 , 3 , 4 , 5

##### Generic diagnosis.

Virgulariid pennatulaceans with polyp leaves rolled or convoluted; 20-26 polyps per polyp leaf; polyp leaves and coenenchyme contain rod-like, somewhat three-flanged sclerites, with parallel sides, broadly triangular at each end, 0.02 to 0.06 mm in length.

##### Type species.

*Grasshoffia virgularioides* by original designation.

##### Etymology.

The genus is named for Dr. Manfred Grasshoff in recognition of his important contributions to the systematics of octocorals, particularly gorgonians and pennatulaceans. He is currently Honorary Scientist at the Senckenberg Research Institute and Natural History Museum, Frankfurt, for his significant contributions to the taxonomy and evolution of octocoral cnidarians. From 1969–2001, Dr. Grasshoff headed the Marine Invertebrates Section at the Institute, where his main research objectives were the taxonomy of octocorals and the evolutionary biology of coelenterates, as well as more general aspects of evolution and phylogeny. From 1972 to 1989 he published several papers on deep-sea pennatulaceans from European and North Atlantic waters (Grasshoff 1972, 1973, 1982).

#### Grasshoffia virgularioides sp. n.

Taxon classificationAnimaliaPennatulaceaVirgulariidae

http://zoobank.org/6D86C220-3DA9-49EE-98E2-E67EEA3290C1

Figures 1 , 2 , 3 , 4 , 5

##### Species diagnosis.

Virgulariid sea pens superficially resembling some species of *Virgularia*. Axis circular in transverse section, extending throughout virtually entire colony length. Polyp leaves variously rolled, funnel-shaped, or semicircular in shape with conspicuous, somewhat narrowed basal stalks. Sclerites of polyps leaves and coenenchyme rod-like with parallel sides and mostly deltoid apices, inconspicuously three-flanged. Preserved colony color cream-white in ethanol.

##### Type material.

Holotype: USNM 1205583, North Atlantic Ocean, Gulf of Guinea, Nigeria, Isaka, Bight of Bonny, Niger Delta, Bonny River; depth not recorded; 28 August 1984; wet-preserved 70% ethanol; one whole colony. Paratype 1: USNM 1231549, same data as holotype; one colony in two pieces 122 mm in total length. Paratype 2: USNM 1231550, same data as holotype; one colony 82 mm in length. Paratype 3: USNM 1205580, same data holotype; one colony in two pieces 119 mm in total length.

##### Description.

*Morphology* (Figures 1–2). The holotype is 98 mm in length. The axis extends throughout the length of the colony and is exposed for 5 mm at the distal-most region of the rachis. The axis is circular to broadly elliptical in cross section (Figure 2C), mottled brown and white in color (presumably due to a mixed content of calcitic and proteinaceous material), composed of narrow wedges of axial material radiating outward from the center, and is approximately 0.80–0.90 mm in diameter. The rachis is also approximately 0.80–0.90 mm in width and is densely congested with polyp leaves; there is approximately 1.5 mm of bare rachis between adjacent proximal bases of the polyp leaves. The polyp leaves are funnel-shaped or mushroom-shaped in lateral view. They broaden distally where the polyps reside, and have neck-like bases that narrow proximally and serve to attach the polyp leaves to the rachis (Figure 2B). The appearance of the polyp leaves are conspicuously rolled or convoluted (some are horseshoe-shaped), with approximately fourteen to twenty-six polyps per leaf (Figures 1A, 2A). The polyps are contractile and non-retractile, urn-shaped or teardrop-shaped and approximately 0.50-0.60 mm in diameter. Siphonozooids not apparent on the rachis or polyp leaves.

*Sclerites* (Figures 3–4). The distal region of the polyp leaves and the tissues of the peduncle contain numerous, small, rod-like sclerites that are prismatically-shaped with straight parallel sides, more-or-less three-flanged, mostly broadly-triangular in shape at each end, and vary in length from 0.02 to 0.06 mm.

*Color* (Figure 1). The color of the wet-preserved colonies is cream-white throughout.

##### Etymology.

The specific epithet is derived from the genus *Virgularia* and the suffix -*oidea* (likeness of form); in reference to the superficial resemblance of the colonies to some species of the genus *Virgularia*.

##### Habitat and distribution.

Habitat not known. Known only from the type locality – Niger River Delta, Nigeria, Gulf of Guinea, West Africa. Depth not recorded (Figure 5).

##### Differential diagnosis.

*Virgularia* and *Scytaliopsis* do not have sclerites in the rachis or polyp leaves. *Scytalium* has ovoid plate-like sclerites in the polyp walls and polyp leaves that are not three-flanged. *Grasshoffia* has prismatically-shaped rod-like sclerites in the polyp leaves and coencenchyme that are indistinctly three-flanged and have broadly-triangular ends. Species of *Stylatula* have a fan-like armature of large spindles at the base of each polyp leaf, and most species of *Acanthoptilum* have a cluster of non-aligned spindles at the base of each polyp leaf. *Virgularia*, *Scytalium*, *Stylatula*, and *Acanthoptilum* generally have flattened polyp leaves that are variously-shaped, while *Grasshoffia* has strongly curved, rolled, often horseshow-shaped to funnel-shaped polyp leaves. *Stylatula macphersoni* López-González, Gili & Williams, 2001, has sclerites in the body walls of the autozooids that are similar in shape to coenenchymal sclerites in *Grasshoffia virgularioides* (López-González et al. 2001; 67, Fig. 4A).

##### Remarks.

The coenenchyme covering the rachis is extremely thin, and therefore the rachis and axis diameters are virtually equal. The polyp leaves are distinctly rolled or conspicuously curved, perhaps due to contraction in the wet preserved type material, as the appearance of the living colonies is not known. Siphonozooids were not observed in the preserved type material, possibly due to the congested and contracted state of the polyp leaves along the rachis.

## Discussion

### Historical perspective

The family name “Virgularidae” was first proposed by Verrill (1868: 382), who included the genera *Virgularia* Lamarck, 1816 and *Stylatula* Verrill, 1864. Subsequently, Kölliker (1869: 123) used the family name “Virgularieae” to include the genera *Virgularia*, *Sylatula*, *Pavonaria* Kölliker, 1869, *Scytalium*, *Acanthoptilum*, *Funiculina* Lamarck, 1816, and *Halipteris* Kölliker, 1866. Gray (1860 and 1870) removed *Funiculina* from the Virgularieae and named the family “Funiculineae” in 1860, changing it to “Funiculinidae” in 1870. Kölliker (1880: 37) defined the family “Virgularidae” to include *Virgularia*, *Dübenia* Koren & Danielssen, 1877, *Stylatula*, *Acanthoptilum*, *Pavonaria*, and *Scytalium*. Jungersen (1904) placed *Pavonaria* in a separate family Pavonaridae. Balss (1910) agreed, but named the family Balticinidae instead of Pavonaridae. Kükenthal and Broch (1911) and Kükenthal (1915) divided the family Virgulariidae into two subfamilies – Pavonariinae to include *Pavonaria*, and Virgulariinae to include *Acanthoptilum*, *Scytaliopsis*, *Scytalium*, *Stylatula*, *Virgularia*. The family Virgulariidae was included by Kükenthal (1915) in the nominal and no longer recognized ranks – Suborder Subselliflorae and Section Pennatulina junciformia, and at the same time the family Halipteridae (Pavonaridae) was treated as a subfamily of the Virgulariidae. The revision of Williams (1995) treated the Halipteridae and Virgulariidae as separate families. Hickson (1916) recognized these two families – Virgulariidae for *Virgularia* and *Stylatula* and Pavonariidae for *Osteocella* Gray, 1870 and *Pavonaria*. Williams (1990, 1995, 2015) recognized *Halipteris* (a genus of seven species) as the valid name for *Balticina* Gray, 1870, *Göndul* Koren & Danielssen, 1883, *Lygomorpha* Koren & Danielssen, 1877, *Microptilum* Kölliker, 1880, *Norticina* Gray, 1870, *Osteocella*, *Pavonaria*, *Stichoptilum* Grieg, 1887, and *Verrillia* Stearns, 1873. Williams (1995: 120) established the family Halipteridae to include the sole genus *Halipteris*, and recognized the Virgulariidae to include the five genera *Acanthopitilum*, *Scytaliopsis*, *Scytalium*, *Stylatula*, and *Virgularia*.

### Taxonomic perspective

The Virgulariidae is here defined as follows (modified from Williams 1990: 86–87, 95 and Williams 1995: 97–98). Pennatulacean octocorals; usually elongate and slender to vermiform, feather-like in appearance in life, usually < 500 mm in length; axis well-developed and present throughout the length of the colony, round to quadrangular in transverse section; proximal portions of adjacent autozooids fused forming conspicuous polyp leaves (flattened expansions, often wing-like) that emanate laterally along the rachis in two opposite longitudinal series; polyp leaves thin and often translucent; polyps without calyces; rachis rod-shaped; peduncle slender and vermiform; siphonozooids present on polyp leaves or on rachis between polyp leaves; sclerites are spindles or rods often three-flanged, plates, minute ovals, or absent. The family is circumglobal in distribution with a depth range of 0-1100 m, and contains six genera – *Acanthoptilum*, *Scytaliopsis*, *Scytalium*, *Stylatula*, *Virgularia*, and *Grasshoffia* gen. n.

### Key to the genera of the family Virgulariidae

**Table:** 

1	Sclerites are present in the rachis and polyp leaves	**2**
–	Sclerites are absent in the rachis and polyp leaves	**5**
2	Sclerites of the polyp leaves are conspicuous spindles or needle-like spindles (0.20–1.50 mm in length)	**3**
–	Sclerites of the polyp leaves are small oval-shaped plates or slightly three-flanged rods (0.02–0.04 mm in length)	**4**
3	Needle-like sclerites form a strong fan-shaped armature at the base of each polyp leaf	***Stylatula***
–	Spindle-like sclerites form a weak cluster at the bases of the polyp leaves (but not as a fan), or they are scattered in the polyp leaves and autozooids (but not as basal clusters)	***Acanthoptilum***
4	Numerous ovoid plate-like sclerites are present in the rachis and polyps leaves; sclerites red in color	***Scytalium***
–	Numerous prismatically-shaped rod-like sclerites, which are indistinctly three-flanged, are present in the polyp leaves and peduncle; sclerites colorless	***Grasshoffia* gen. n.** (Figs 1–4)
5	The polyps of a single polyp leaf are of equal size; number of polyps per polyp leaf are highly variable (3–100 or more)	***Virgularia***
–	The polyps on the inner portion of a single polyp leaf are smaller in size than those of the outer portion of the leaf; polyps per polyp leaf are few (4–7 in number)	***Scytaliopsis***

## Supplementary Material

XML Treatment for Grasshoffia

XML Treatment for Grasshoffia virgularioides
